# MDM2-Mediated Degradation of p14ARF: A Novel Mechanism to Control ARF Levels in Cancer Cells

**DOI:** 10.1371/journal.pone.0117252

**Published:** 2015-02-27

**Authors:** Maria Vivo, Maria Matarese, Maria Sepe, Rosaria Di Martino, Luisa Festa, Viola Calabrò, Girolama La Mantia, Alessandra Pollice

**Affiliations:** 1 Dipartimento di Biologia, Università di Napoli Federico II, Naples, Italy; 2 Istituto di Genetica e Biofisica (IGB)—Consiglio Nazionale delle Ricerche (CNR), Naples, Italy; 3 Dipartimento di Medicina Molecolare e Biotecnologie Mediche- Università di Napoli Federico II, Naples, Italy; 4 Istituto di Biochimica delle Proteine (IBP)—Consiglio Nazionale delle Ricerche (CNR), Naples, Italy; 5 Diagnostica e Farmaceutica Molecolare- Consiglio Nazionale delle Ricerche (CNR), Naples, Italy; German Cancer Research Center, GERMANY

## Abstract

We here show a new relationship between the human p14ARF oncosuppressor and the MDM2 oncoprotein. MDM2 overexpression in various cancer cell lines causes p14ARF reduction inducing its degradation through the proteasome. The effect does not require the ubiquitin ligase activity of MDM2 and preferentially occurs in the cytoplasm. Interestingly, treatment with inhibitors of the PKC (Protein Kinase C) pathway and use of p14ARF phosphorylation mutants indicate that ARF phosphorylation could play a role in MDM2 mediated ARF degradation reinforcing our previous observations that ARF phosphorylation influences its stability and biological activity. Our study uncovers a new potentially important mechanism through which ARF and MDM2 can counterbalance each other during the tumorigenic process.

## Introduction

Knowledge of the mechanisms governing the unbalance between tumor suppressors and oncogenes, is critical to understanding the pathogenesis and evolution of cancer.

Among the most important tumor suppressors, p14ARF (mouse p19ARF) (Alternative Reading Frame) appears to play a major role, being its direct contribution to cancer largely demonstrated over the last years [1,2].

ARF is involved in oncogenic checkpoint by sensitizing incipient cancer cells to undergo growth arrest or apoptosis. There is growing evidence that ARF signaling is complex, and involves p53-dependent or independent pathways aiming mainly at restraining abnormal cell growth and at maintaining genomic stability. The discovery of a plethora of ARF interactors and the observation that also viral, genotoxic, hypoxic and oxidative stresses activate an ARF response, suggest that ARF has a wider role to protect the cell [3]. Primary cells in normal conditions maintain ARF at low levels; however, when cells are stimulated by oncogenic insults, its concentration in the cell drastically increases. This phenomenon is generally accompanied by a parallel disruption of the inhibitory interaction between Mdm2 and p53, resulting in the accumulation of transcriptionally active p53 that stimulates either apoptosis or cell cycle arrest [4, 5]. The strong ARF effect on cell proliferation requires that cells should have developed mechanisms that can promptly reduce ARF intracellular levels when its activity is no more required. However, the mechanisms that regulate ARF turnover are only recently going to be elucidated. ARF degradation depends, at least in part, on the proteasome and, although ARF lacks lysine residues in its sequence, it can undergo N-terminal ubiquitination independently of Mdm2 and p53 [6]. Quite recently, a specific ubiquitin ligase for ARF called ULF was identified [7]. On the other hand, there are evidences that ARF can be degraded *in vitro* by the 20S proteasome in the absence of ubiquitination and that this process can be counteracted by TBP-1 (Tat Binding Protein 1), a multifunctional component of the regulatory subunit of the proteasome [8]. Furthermore, the REG γ proteasome has been implicated in the ubiquitin-independent regulation of p19ARF turnover, supporting the notion that ubiquitination could be not necessarily implicated in ARF turnover [9].

Interestingly, we and others recently demonstrated that, following PKC (Protein Kinase C alpha) activation, ARF levels increase [10, 11]. Furthermore, a point mutation that mimicks phosphorylation in the conserved Thr8 induces ARF accumulation mainly in the cytoplasm and inhibits its biological activity [11].

So far, ARF subcellular localization appears to play an important role in its stability and biological functions, although in not unequivocal manner. It appears that nucleolar localization of ARF may serve for its storage or stabilization [12,13]. In the nucleolus, ARF assumes a stable structure thanks to its association to B23/NPM, while in the nucleoplasm it is subjected to a more rapid turnover. In some cases, the increase in ARF levels causes Mdm2 to relocate to the nucleolus [14, 15] and this has been linked to p53 stabilization. Others reported that, although nucleolar localization of ARF causes its stabilization, this is not essential to regulate ARF’s activity towards p53 [16, 17]. Interestingly, it has been reported that non-nucleolar forms of ARF are subjected to rapid degradation by the proteasome, with MDM2 playing a role in the modulation of this phenomenon although with mechanisms far from being fully elucidated [18]. MDM2 has multifaceted roles in protein degradation. In fact, aside its well-described role as E3-ubiquitin ligase, MDM2 is able to shuttle p63 to the cytoplasm mediating its interaction with proteins specifically involved in its turnover [19]. Moreover, MDM2 has been shown to mediate proteasome-dependent but ubiquitin-independent degradation of p21Waf1/Cip1 [20] and of Retinoblastoma proteins [21]. More recently it has been reported that MDM2 can interacts with components of the 19S proteasome [22] claiming a wider view of its mechanism of action. We here investigate on a new interrelationship between ARF and Mdm2 in which Mdm2 appears to be implicated in the regulation of ARF turnover mediating its degradation through the proteasome.

## Results

### Mdm2 overexpression causes p14ARF degradation through the proteasome

**Fig 1:**
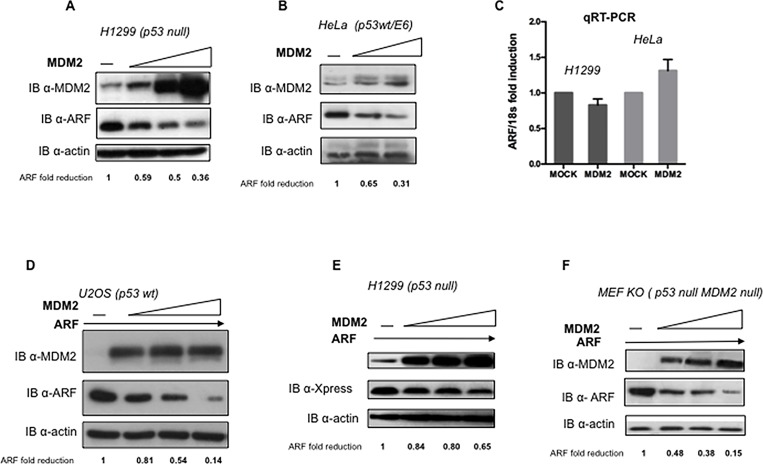
MDM2 overexpression causes reduction of p14ARF levels. **A** H1299 cells were mock transfected (first lane) or transfected with increasing amounts of MDM2 expression plasmid (0.3-0.6-0.9 μg). Effect on p14ARF intracellular levels was evaluated by Western Blot on whole protein lysates probed with anti-ARF and, as control, with anti-MDM2 and anti-actin. **B** HeLa cells were mock transfected or transfected with MDM2 expression plasmid (0.3–0.6 μg) and analysed as in *A*. **C** Real Time relative quantification of ARF expression in H1299 and HeLa cells. Cells were transfected by electroporation with MDM2 plasmid (4x10^6^ cells/2.5 μg of MDM2 expression plasmid) and subjected to RNA extraction and qRT-PCR analysis. ARF RNA levels were normalized respect to 18s RNA. Values in the graph represents the average of three independent experiments. Standard deviation are shown. **D** U2OS, **E** H1299 and **F** MEF p53^-/-^ MDM2^-/-^ cells were transfected with a fixed amount of p14ARF expressing plasmid (0.3 μg first lane) alone or with increasing amounts of MDM2 expressing plasmid (0.3-0.6-0.9 μg). Effect on p14ARF levels was evaluated by Western Blot on whole protein lysates, probed with anti-ARF or anti-Xpress (to detect exogenously expressed ARF) and, as control, with anti-MDM2 and anti-actin. Western Blot shown are representative of at least three independent experiments. ARF band intensities were quantified by Image J software, actin normalized and expressed as fold enrichment respect to untreated samples arbitrarily set to 1 (See Materials and Methods for details).

**Fig 2:**
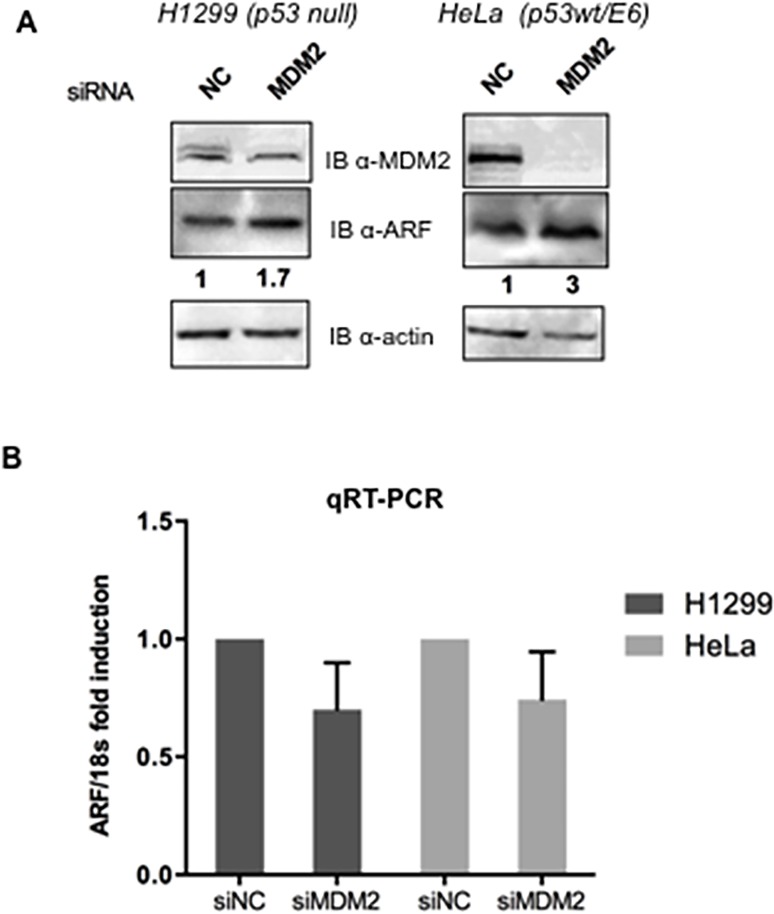
MDM2 silencing causes increase of endogenous ARF levels. H1299 and HeLa cells were treated with either MDM2 or Negative Control directed siRNA (10 nM final concentration) and, after 72 hours of incubation, collected and analysed by Western blot and and qRT-PCR as described in Materials and Methods.

**Fig 3:**
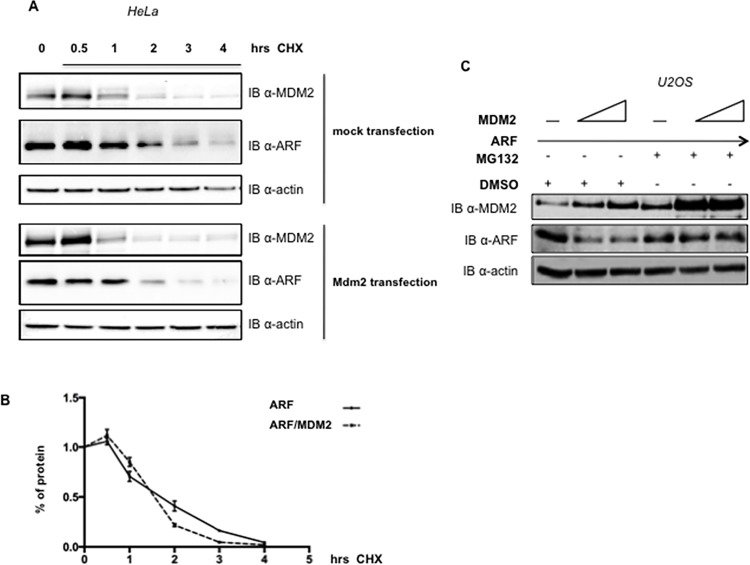
p14ARF turnover analysis. **A** Half-life analysis in presence or absence of MDM2 overexpression. HeLa cells were mock transfected or transfected with MDM2 expression plasmid (0.6 μg). 24 hours after, cycloheximide was added and cells were harvested at the indicated time points. Lysates were analysed by Western Blot with anti-ARF, anti-actin and anti-MDM2. **B** The plot represents half-life analysis of ARF in presence or absence of MDM2 overexpression. Band intensities were quantified by Image J software and actin normalized before being plotted in graph. The amount of protein at different time points is expressed as percentage of total protein, i.e. protein amount at t0. Each profile represents the mean of three independent transfections and Western Blot experiments. Standard deviations are also shown. **C** U2OS cells were transfected with a fixed amount of p14ARF expression plasmid (0.3 μg) and increasing amounts of MDM2 expression plasmid where indicated (0.3–0.6 μg). 24 hours after transfection, cells were treated with 10 μM MG132 (lanes 4–6) or DMSO (lanes 1–3). Levels of p14ARF were analysed by Western Blot on whole protein lysates, probed with anti-ARF and, as control, with anti-MDM2 and anti-actin.

To analyze the potential involvement of MDM2 in the regulation of p14ARF protein stability we overexpressed a MDM2 expression plasmid in different human and mouse cell lines that present or lack detectable levels of p53 and/or MDM2. As Fig. 1 shows, p14ARF endogenous levels in both H1299 and HeLa cells linearly decrease when increasing amounts of MDM2 expression plasmid were transfected (Fig. 1, A and B). Real-Time PCR analysis on RNA extracted from MDM2 transfected HeLa and H1299 cells shows no significant changes in the relative amount of ARF mRNA (Fig. 1C) indicating that the MDM2 effect is exerted at the post-transcriptional level. Furthermore, MDM2 overexpression results also in reduction of exogenously expressed ARF protein in several human and mouse cell lines (Fig. 1D, E and F) irrespective of the p53 status, indicating that MDM2 affects ARF protein stability in a p53-independent way. Importantly, reduction of endogenous MDM2 intracellular levels by siRNA causes an increase of ARF endogenous levels in both HeLa and H1299 cells, further confirming that endogenous MDM2 can control p14ARF abundance (Fig. 2). We thus investigated ARF protein stability in the presence or absence of MDM2 overexpression following exposure to cycloheximide to block protein synthesis. At the indicated times after exposure to the drug, cells were harvested and the extracts were analyzed by Western Blot. Fig. 3A and B shows that endogenous p14ARF half-life is lowered following MDM2 overexpression.

We next asked whether the proteasome is involved in MDM2-mediated p14ARF degradation. To explore this hypothesis, we transfected U2OS cells with a fixed amount of p14ARF and increasing amounts of MDM2 and treated cells with the proteasome inhibitor MG132. Western Blot in Fig. 3C consistently shows that treatment with the proteasome inhibitor counteracts the MDM2 effect, indicating the proteasome as the final effector of the MDM2 action on ARF.

### ARF degradation by MDM2 requires protein-protein interaction

**Fig 4:**
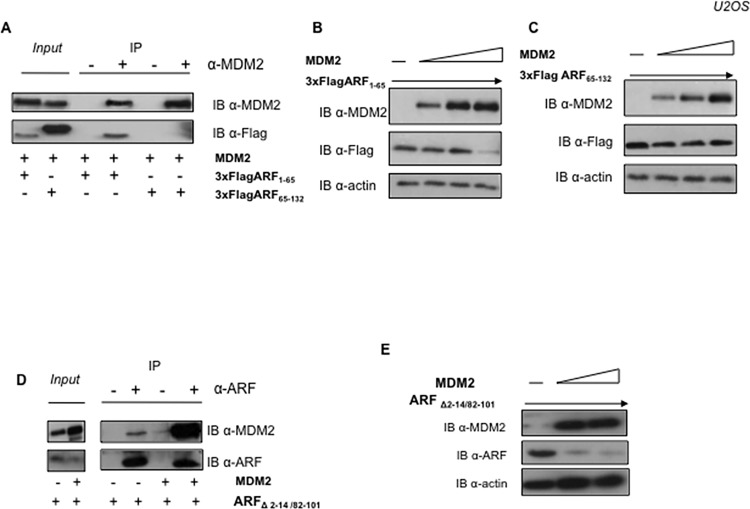
ARF degradation by MDM2 requires protein-protein interaction. **A** U2OS cells were transfected with equal amount (1 μg) of MDM2 and either 3xFlagARF_1–65_ or 3xFlagARF_65–132_ expressing plasmids, as indicated. Equal amounts of protein extract were immunoprecipitated with anti-MDM2 antibody and revealed by Western Blot with anti-MDM2 and anti-Flag to reveal ARF. **B** U2OS cells were transfected with 0.3 μg of 3xFlagARF_1–65_ (expressing exon 1β of p14ARF) (first lane) alone or with increasing amounts of MDM2 expression plasmid (0.3-0.6-0.9 μg). Levels of p14ARF were analysed by Western Blot on whole protein lysates, probed with anti-ARF and, as control, with anti-MDM2 and anti-actin. **C** U2OS cells were transfected with 0.3 μg of 3xFlag ARF_65–132_ plasmid (expressing exon 2 of p14ARF) (first lane) alone or with increasing amounts of MDM2 expression plasmid (0.3-0.6-0.9 μg). Levels of p14ARF were analysed by Western Blot on whole protein lysates probed with anti-ARF and, as control, with anti-MDM2 and anti-actin. **D** U2OS cells were transfected with 1 μg of both ARFΔ_2-14/82-101_ and MDM2 expression plasmid as indicated. Equal amounts of protein extract were immunoprecipitated with anti-ARF antibody and revealed by Western Blot with anti-MDM2 and anti-ARF. **E** U2OS cells were transfected with 0.3 μg ARFΔ_2-14/82-101_ and increasing amounts of MDM2 expression plasmid where indicated (0.6–0.9 μg). Levels of endogenous p14ARF were analysed by Western Blot on whole protein lysates probed with anti-ARF and, as control, with anti-MDM2 and anti-actin.

The physical interaction between p14ARF and MDM2 has been the object of intensive studies and multiple interacting domains have been identified in p14ARF that contribute to the binding to MDM2 [14, 17, 23, 24]. Hence, we decided to analyze which regions of ARF are necessary to observe the MDM2 effect on p14ARF. We thus made use of two different ARF deletion mutants, the ARF_1–65_, encompassing exon 1β of p14ARF and the ARF_65–132_, corresponding to ARF exon 2. Co-immunoprecipitation in U2OS cells showed that the ARF_1–65_ mutant is able to interact with MDM2 while that retaining only exon 2 (ARF_65–132_) is not (Fig. 4A), confirming a major role of exon 1β in binding MDM2 [23, 24]. The two ARF deletion mutants were then transfected together with increasing amounts of MDM2. As Fig. 4B and C clearly show, the amino terminal half of p14ARF (ARF_1–65_) appears degraded by MDM2 overexpression, while the C-terminal half (ARF_65–132_) is not affected, supporting a role of the binding between the proteins in the MDM2 mediated ARF degradation.

Further, we analyzed the capacity of MDM2 to induce degradation of an ARF deletion mutant (ARFΔ_2-14/82-101_) lacking the nuclear/nucleolar localization signals [14]. Interestingly, this mutant displays a prevalent cytoplasmic localization [14] and retains the capacity to bind (Fig. 4D) and be degraded by MDM2 (Fig. 4E).

**Fig 5:**
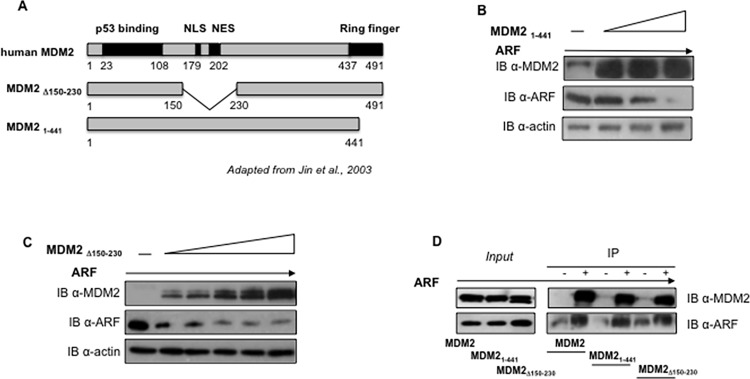
ARF degradation by MDM2 does not require the ubiquitin ligase domain of MDM2 nor its nuclear/nucleolar localization. **A** The scheme indicates MDM2 mutants described in this figure [25]. **B** U2OS cells were transfected with a fixed amount of p14ARF expressing plasmid (0.3 μg) and increasing amounts of MDM2_1–441_ expressing plasmid (0.3-0.6-0.9 μg). Levels of p14ARF were analysed by Western Blot on whole protein lysates probed with anti-ARF and, as control, with anti-MDM2 and anti-actin. **C** U2OS cells were transfected with a fixed amount of p14ARF expressing plasmid (0.1 μg) and increasing amounts of MDM2Δ_150–230_ expressing plasmid (0.5-1-1.5-2-2,5-3 μg). Levels of p14ARF were analysed by Western Blot on whole protein lysates probed with anti-ARF and, as control, with anti-MDM2 and anti-actin. **D** U2OS cells were transfected with equal amount (1 μg) of p14ARF expressing plasmid and MDM2_1–441_ or MDM2Δ_150–230_ expressing plasmid. Equal amounts of protein extract were immunoprecipitated with anti-ARF antibody and revealed by Western Blot with anti-MDM2 and anti-ARF.

Aside the well established role as an ubiquitin ligase, MDM2 can affect protein turnover also through an ubiquitin independent mechanism, inducing a direct association of target proteins with the proteasome [22]. To discriminate between these possibilities, we analyzed the effect of a MDM2 mutant lacking the ubiquitin ligase ring finger domain (MDM2_1–441_) (Fig. 5A) [25] on exogenously expressed ARF levels. Interestingly, this mutant is able to induce ARF degradation when overexpressed (Fig. 5B), indicating that the MDM2 ubiquitin ligase domain of MDM2 is dispensable for the observed effect on ARF. Next, we analyzed the effect of a MDM2 deletion mutant (MDM2Δ_150–230_) (Fig. 5A) that, lacking both the import (NLS) and export (NES) nuclear localization signals, shows a prevalent cytoplasmic localization [25]. When overexpressed in U2OS cells, this mutant induces degradation of exogenously expressed ARF (Fig. 5C) indicating that ARF degradation does not require MDM2 localization in the nucleus. Co-immunoprecipitation experiments indicate that both MDM2 mutants are able to bind ARF, reinforcing the notion that the binding between the two proteins could play a role in the observed effect (Fig. 5D).

### The MDM2-mediated ARF degradation mainly occurs in the cytoplasm

**Fig 6:**
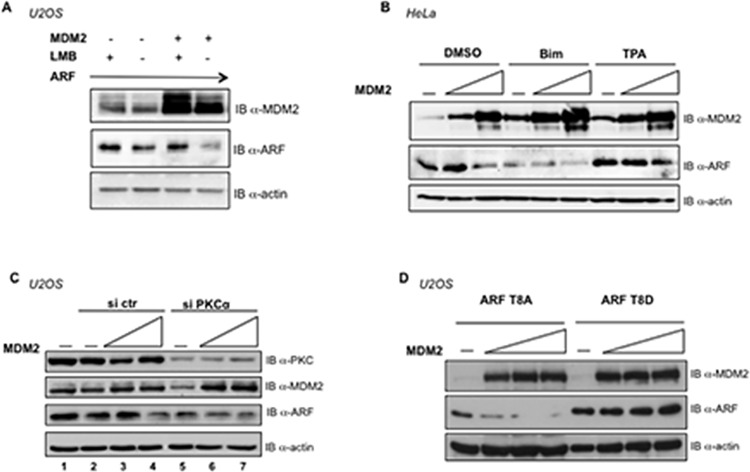
ARF subcellular localization and phosphorylation status influences its turnover. **A** U2OS cells were transfected with 0.5 μg of ARF expression plasmid alone (lanes 1, 2) or in combination with 1 μg of MDM2 expression plasmid (lanes 3, 4). 24 hours after transfection cells were treated with Leptomycin B (lanes 1 and 3) or Methanol (lanes 2 and 4). Total cell extracts have been subjected to Western Blot and analyzed with anti-ARF, anti-MDM2 and anti-actin antibodies. **B** HeLa cells were transfected with increasing amounts of MDM2 expression plasmid (1 and 1.5 μg). 24 hours after transfection, cells were treated with either DMSO, TPA or Bisindolylmalemide and analyzed with anti-ARF, anti-MDM2 and anti-actin antibodies in Western Blot. **C** HeLa cells were transfected with PKCα siRNA or negative control siRNA (siRNActrl). 48 hours later, cells were mock transfected or transfected with increasing amounts of MDM2 expressing plasmid where indicated (0.6 and 0.8 μg plasmid DNA). Total cell extracts were analyzed by Western blot with anti ARF, anti-MDM2 and anti-actin antibodies. **D** U2OS cells were transfected with 0.3 μg of either ARFT8A or ARFT8D alone (first lanes) or in combination with increasing amounts of MDM2 expression plasmids (0.3-0.6-0.9 μg). 24 hours after transfection cell extracts were subjected to Western Blot with anti ARF, anti-MDM2 and anti-actin.

The evidence that a MDM2 mutant, unable to localize in the nucleus, induces ARF degradation, together with the observation that an ARF mutant displaying a predominant cytoplasmic localization (ARFΔ_2-14/82-101_) is prone to MDM2-mediated degradation, lead to the suggestion that MDM2 induced ARF degradation upon MDM2 overexpression can occur in the cytoplasm. This prompted us to verify the ability of MDM2 to degrade ARF in cells treated with Leptomycin B (LMB), known to block MDM2 shuttling to the cytoplasm [26]. To this purpose, U2OS cells transfected with a fixed amount of ARF were treated or not with Leptomycin B in presence or absence of ectopic MDM2. As shown in Fig. 6A, Leptomycin B treatment not only determines accumulation of ARF but, more importantly, counteracts the MDM2 effect on ARF, strongly indicating that forced nuclear MDM2 localization prevents its ability to affect p14ARF turnover.

### MDM2 mediated ARF degradation is prevented upon PKC activation.

We recently demonstrated that TPA (a known activator of the signal transduction enzyme PKCα) treatment results in an increase of the ARF cytoplasmic pool [11]. Here we addressed whether PKC activation could influence MDM2-mediated ARF degradation. To this purpose we treated HeLa cells, transfected with increasing amounts of MDM2, with either TPA or Bisindolylmalemide (Bim, an inhibitor of PKC activation). Fig. 6B shows that Bim treatment, as expected, induces a decrease of ARF. Interestingly, MDM2 overexpression further lowers ARF levels compared to controls. Conversely, TPA treatment mostly counteracts MDM2 induced degradation of p14ARF, suggesting that the PKC pathway should not be activated to obtain efficient MDM2-mediated ARF degradation. To further analyse the PKC role in MDM2 mediated ARF degradation, U2OS cells were treated either with PKC α specific siRNA or control siRNA and transfected with increasing amounts of MDM2 (Fig. 6C). Overexpression of MDM2 in si-ctr depleted cells induces ARF degradation as expected. Interestingly, we observe a decrease of ARF levels upon PKC depletion in the absence of MDM2 overexpression (compare lane 5 with lane 2). This is in agreement with the observation that PKC activation induces an increase of ARF protein levels and PKC inactivation causes a decrease of ARF levels (Vivo et al., 2013 and Fig. 6B). We actually observed that also MDM2 levels are negatively regulated by PKC depletion, (compare lane 5 with lane 2). Importantly, in agreement with our previous experiments, MDM2 overexpression in PKC depleted cells can still reduce ARF protein levels (compare lane 5 with lanes 6 and 7).

To go deeper inside the potential role of ARF phosphorylation in MDM2 mediated ARF degradation, we made use of two point mutants, the ARFT8D and ARFT8A in which Threonine 8 has been substituted, in order to mimic, respectively, a phosphorylated and a non phosphorylated form of ARF [11]. Interestingly, the dephosphorylated form of ARF (T8A mutant) is very unstable, with a relatively short half-life while the T8D appears consistently enriched in the cytoplasmic compartment [11].

Both mutants were transfected in U2OS cells together with increasing amounts of MDM2. Interestingly, the phospho-mimetic T8D mutant appears more resistant to MDM2 mediated degradation, while the T8A mutant appears more sensible to the MDM2 effect (Figs. 6D and 1D), confirming that phosphorylation exerts a protective role on ARF.

**Fig 7:**
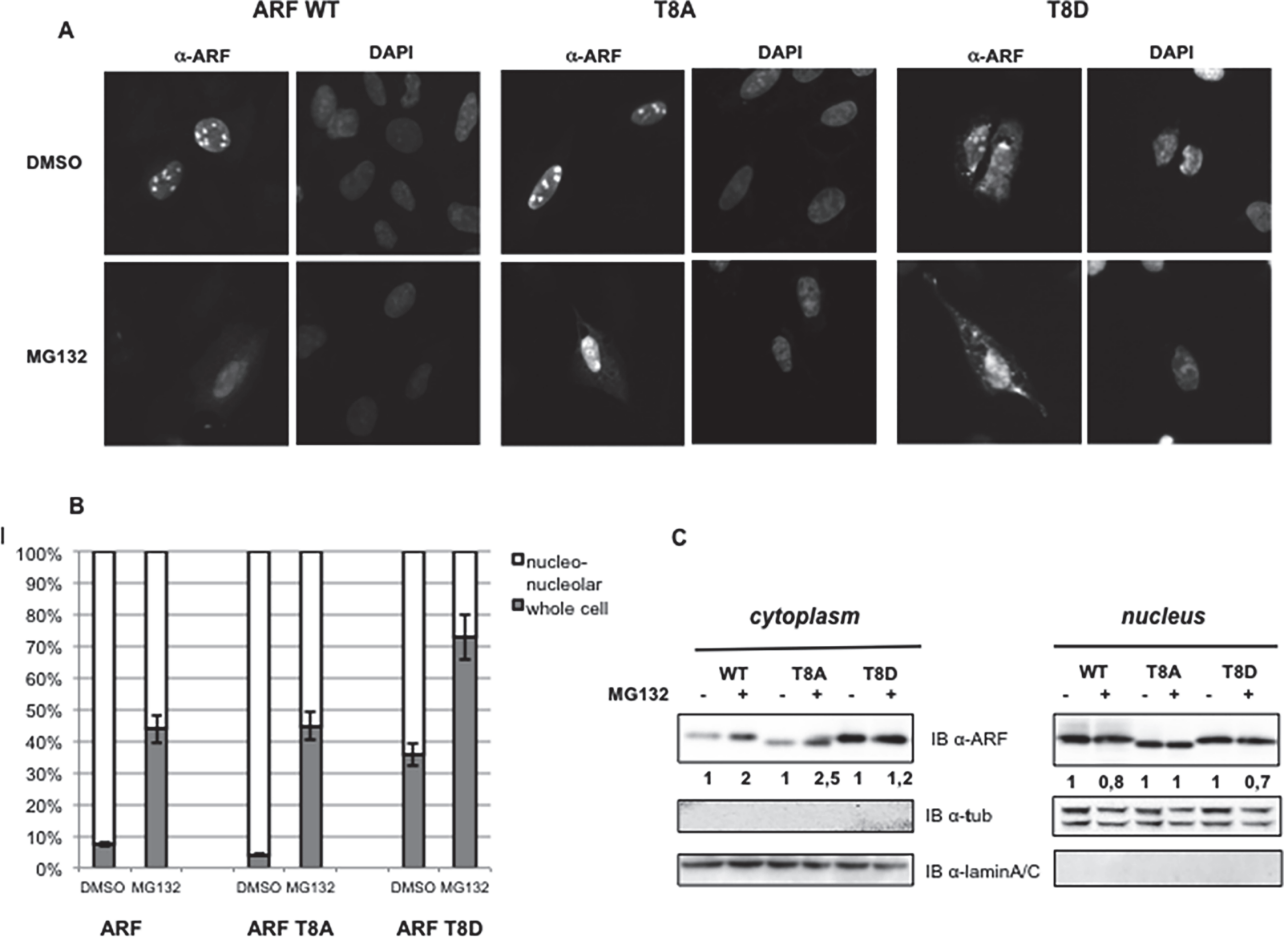
ARF accumulates in the cytoplasm upon proteasome inhibition. **A** U2OS cells were transfected with 1,5 μg of either wt or mutants ARF expressing plasmids. 16 hours upon transfection, cells were treated with either DMSO or 10 μM MG132 for 5 hours and subjected to IF with anti-ARF antibody. Representative images showing ARF and ARF mutants subcellular localization in U2OS cells with or without MG132 treatment are shown. Images were taken with a Nikon fluorescence microscope at similar exposure conditions. **B** The histogram, representing the mean of three independent experiments, reports the percentage of transfected cells showing each localization pattern. Standard deviations are also shown. **C** Western blot analysis of equal amounts of both cytoplasmic (30 μg) and nuclear (6 μg) protein extracts of transfected U2OS cells treated or not with MG132. Efficiency of cellular fractionation was checked with anti-lamin A/C and anti-tubulin antibodies. Normalized ARF band intensities, shown below each corresponding band, are expressed as fold enrichment respect to untreated samples arbitrarily set to 1 (see Materials and Methods for details).

This result raised the hypothesis that the prevalent nuclear localization of ARF (T8A and WT proteins) could be the result of an efficient clearance of dephosphorylated, and thus unstable, ARF protein from the cytoplasm. To explore this hypothesis, U2OS cells, transfected with wild type or mutant proteins were treated with MG132 and ARF subcellular distribution was analyzed by immunofluorescence. As is shown in Fig. 7A and B we observed, upon MG132 treatment, a consistent increase of ARF cytoplasmic staining in both WT and T8A transfected cells, further suggesting that ARF degradation prevalently occurs in the cytoplasm. Similarly, Western Blot of nuclear and cytoplasmic extracts of U20S cells, transfected and treated as above, shows a consistent increase of both wt ARF and ARFT8A protein levels in the cytoplasmic compartment upon proteasome inhibition (Fig. 7C).

## Discussion

The importance of the functional interaction between ARF and MDM2 came out from the initial discovery that ARF has the potential to act as a tumour suppressor by binding to and inhibiting the p53 antagonist MDM2. This knowledge was reinforced and refined over years although with controversies regarding the role of ARF nucleolar localization in this process [16, 17, 27]. On the other hand, the importance of this interaction is clear from the observation that inactivation/deregulation of the p53-MDM2-ARF axis is crucial in the development of most human cancers. Intriguingly, MDM2 overexpression and p14ARF loss correlate with a more aggressive phenotype in primary human lung tumors underlying an inverse relationship between the two in human tumors [28]. On the other hand, previous observations pointed to a controversial effect of MDM2 action on p14ARF: it was shown that loss of Mdm2 could either suppress [29] or restore [14] the ability of murine p19 ARF to induce p53-independent cell cycle arrest. Thus, depending on the context (cellular types, upstream signals, expression level), Mdm2 could behave either as a mediator or inhibitor of p14ARF function onto cell proliferation [28]. Anyway, the first “upturn” in our way to look at the MDM2/ARF relationship came out from the observation that a short ARF mutant (aa2-29) appeared significantly stabilized after silencing of MDM2 or overexpression of MDMX (a MDM2 related, interfering protein), leading to a model in which ARF binding to MDM2 causes, in some way, its suicide because brings itself to degradation (“guilt by association model”) [18]. Our study well fit in with these observations, demonstrating that MDM2 intracellular increase can, indeed, cause ARF destruction by the proteasome. We demonstrate that the physical interaction between ARF and MDM2 is necessary, although not sufficient in order to observe the effect, as ARFT8D mutant is able to interact with MDM2 [11] but is not degraded. The fact that the region of interaction of MDM2 with MDMX (i.e the RING Finger domain) is dispensable for this effect suggests that, indeed, this protein is not involved. On the other hand, the observation that the RING Finger/Ubiquitin ligase domain of MDM2 is dispensable to affect ARF levels, suggests that, although this cannot be formally excluded, ARF ubiquitination is not required [8, 9]. On the other hand, Rodway et al. [18] postulated a role of MDM2 in mediating ARF delivery to the proteasome without any requirement for ubiquitination. This could probably be mediated by MDM2 direct interaction with the proteasome [22]. In particular, Mdm2 has been shown to directly interact with several proteasome subunits through both N-terminal and C-terminal regions while its central domain (aa200-300) appears to exert a negative regulative control on the binding [22]. Our MDM2 mutants (MDM2_1–441_ and MDM2Δ_150–230_) retain at least one of the regions required for the interaction with the proteasome and both of them are able to induce ARF degradation. Intriguingly, we also observe a more efficient ARF reduction with the MDM2Δ_150–230_ mutant, that lacks the central domain.

However, as ARF ubiquitination has not been directly addressed in the present study, we cannot exclude the intervention of ULF [7] or any other yet unidentified ubiquitin ligase in the observed effect. However, while ULF mediated ARF degradation appears exerted in the nucleoplasm, different observations in this paper indicate that the MDM2 effect occurs in the cytoplasm. Firstly, mutations in both ARF and MDM2 that force their localization in the cytoplasm, do not affect MDM2 mediated ARF degradation. Furthermore, Leptomycin B treatment, that inhibits MDM2 nuclear export, causes ARF accumulation and counteracts the MDM2 degradation effect on ARF. Finally, immunofluorescence experiments clearly show that MG132 treatment results in an increase of ARF levels in the cytoplasm, strongly suggesting that, indeed, the reason why ARF is not usually detectable in this subcellular compartment resides in a more rapid turnover.

It has to be remarked that, since its initial discovery, ARF was described with a prevalent nucleo/nucleolar localization. Accordingly, it has been reported that a protective role on ARF levels is exerted in the nucleolus by B23/NPM. Moreover, interaction with TBP1, which protects ARF from proteasome degradation, occurs in the nucleus [8].

More recently, ARF has been reported to localize also in the cytoplasm although mainly associated to mitochondria, because of its role in autophagy [30]. Interestingly, cytoplasmic p14ARF staining has been described in some cancers [28].

Finally, we have recently reported that, following activation of PKC, ARF protein is phosphorylated and accumulates in the cytoplasm [11].

Here we show that activation of the PKC pathway by TPA counteracts MDM2 action while inhibition of the pathway by either Bisindolylmalemide treatment or PKCα directed siRNA, causes a decrease of ARF levels and enhances MDM2 mediated ARF degradation. Accordingly, the T8D ARF mutant that mimics the phosphorylated status of the protein, although more present in the cytoplasm, appears to be less sensitive to MG132 treatment and to MDM2 overexpression. These data reinforce our previous suggestion that phosphorylation exerts a protective effect on ARF. It seems that more than one transcription independent mechanisms are involved in the regulation of ARF protein, i.e. proteasome mediated degradation [6–8] and phosphorylation.

Overall, our observations lead to the hypothesis that in an oncogenic environment in which MDM2 is overexpressed, ARF levels are kept low due to a rapid turnover caused by MDM2 triggered degradation. Increasing ARF degradation and/or phosphorylation could thus be common strategies that a cell orchestrate to promptly escape ARF growth suppression functions either in physiological conditions or when cells are forced to proliferate during cancer progression. Further experiments are needed to clarify the molecular mechanisms underlining these observations and their relevance for cancer development *in vivo*.

## Materials and Methods

### Constructs

pcDNARF, 3xFlag ARF_1–65_, 3xFlagARF_65–132_, ARFΔ_2-14/82-101_ were described in [8]. pcDNAARFT8A and T8D plasmids were described in [11]. pCMVMDM2 wt, pCMVMDM2_1–441_, pCMVMDM2Δ_150–230_ were described in [31].

### Cell cultures, transfections

U2OS, H1299, Hela cell lines were purchased from the Cell Lines Service (CLS). *MEF* ^*p53-/-MDM2-/-*^ cell line was described in [32]. All cell lines were grown in Dulbecco’s modified Eagle medium supplemented with 10% fetal bovine serum (Euroclone, Life Science) at 37°C in a humidified atmosphere of 5% (v/v) CO2 in air.

Cells were transfected using Lipofectamine 2000 (Life Technology) [33] or with RNAiMAX (Life Technology) [34] according to the manufacturer’s recommendations.

For experiments described in Fig. 7C, U2OS cells were transfected by electroporation using the Neon Transfection System (Life Technology) following manufacturer instructions. Briefly, 4x10^6^ cells were electroporated with 2.5 μg of either wt ARF or T8D ARF mutant plasmid DNA, and 3.5 μg of ARF T8A expressing plasmid. In order to achieve the same transfection efficiency in untreated and MG132 treated samples, transfected cells were splitted in two aliquots and, 24 hours post transfection, incubated with either DMSO or MG132.

### Real Time PCR Analysis

Total cell extracts from HeLa cells were collected for each experimental point using the PureLink RNA Mini Kit-Life Technology according to manufacturer instructions. cDNA synthesis was performed using 1ug of total RNA with SuperScript III RT (Life Technology). Real Time PCR was performed using 50 ng of ss cDNA with Syber Green PCR master mix (Applied Biosystems). Relative quantification of p14ARF transcripts was done using 18s RNA for normalization.

*Oligo sequences*:

p14ARF For: 5’CCTCGTGCTGATGCTACTGAGGGACCAGCGTCTAGG3’

p14ARF Rev: 5’ ACGTCCGCCACCCGGGCGCT 3’

18S For: 5’tcgaggccctgtaattggaa3’

18S Rev: 5’ CTTTAATATACGCTATTGGAGCTGGAA3’

### Decay rate analysis

Decay rate analysis was already described [33]. Briefly, HeLa cells were either mock transfected or transfected with pCMVMDM2. Twenty-four hours after transfection, cycloheximide was added at a final concentration of 60 μg /ml, cells were harvested at the indicated time points and extracts analyzed by Western Blot with anti-ARF (Clone C-18, Santa Cruz), anti MDM2 (clone 2A10, Calbiochem) and anti actin (Clone I-19, Santa Cruz). Band intensities at the different time points were quantified by Image J Software, normalized to actin and reported in graph as percentage of total protein (protein a t0). Each profile represents the mean of three independent experiments. Standard deviations are also shown.

### Silencing experiment

H1299 and HeLa cells were plated in six-well plates at 2,5*10^5^ cells/well. 24 hours later, cells were transfected with either with 10 pmoles of siRNA targeting MDM2 or siNegative Control (Qiagen) (10nM final siRNA concentration). Cells were harvested 72 hours later and processed as described before.

U2OS cells were plated in 24 well plates at 5*10^4^ cells/well. 24 hours later, cells were transfected with either with 15 pmoles of siRNA targeting PKCα or siNegative Control [33]. SiRNA were purchased from Qiagen (Flexi Tubes). 48 hours later, cells were mock transfected (lane 1) or transfected with 0.3 μg of ARF alone or in combination with increasing amounts of pCMVMDM2 expressing plasmid (0.6 and 0.8 μg). Twenty-four hours later cells were collected and total cell extracts analyzed by Western Blot.

### Immunoblot analyses, Co-immunoprecipitation, subcellular fractionation

Western Blot analysis was performed as previously described [35]. Extracts were blotted onto PVDF Immobilon-P transfer membrane (Millipore cat. NO IPVH00010). Proteins were visualized with an enhanced chemi-luminescence images detection system (Thermo Scientific ECLplus) and images were taken with ChemiDoc XRS System (Bio-Rad Laboratories). Image quantification was performed with Image J analysis software as described above.

*CoIPs* were performed as described [32]. Briefly, lysates from cells transfected with 1:1 ratio of either pCDNA-ARF and various MDM2 constructs or pCMV MDM2 and various ARF constructs (as described in the text) were incubated with anti-ARF, or with anti-MDM2 antibodies.

Antibodies used in this study: anti-ARF (C-18 clone, Santa Cruz), anti-ARF (14PO2 clone, Neomarkers), anti-actin (I-19 clone, Santa Cruz), anti-MDM2 (2A10 clone, Calbiochem); anti X-press monoclonal antibody (Life Technology); anti-Flag (M2 clone, Sigma-Aldrich), anti-β-tubulin (clone H235, Santa Cruz), anti lamin A/C (clone H110, Santa Cruz).

### Treatments in this study were performed as follows

For the treatment with MG132 proteasome inhibitor: U2OS cells were treated either with DMSO or 10 μM MG132 (Sigma-Aldrich) for 5 hours. Cells were harvested and total cell extracts were prepared for subsequent analysis as described [8].

Treatment with Bisindolylmalemide or TPA was performed as follows: 24 hours after transfection, HeLa cells were treated either with DMSO or Bisindolylmalemide (Calbiochem) at 10 μM final concentration for 16 hours or TPA (Applichem) at 10 μM final concentration for 5 minutes. Cells were harvested and total extracts prepared for subsequent analysis as described.

Treatment with Leptomycin B: 24 hours after transfection, U2OS cells were treated either with Methanol or with Leptomycin B (Sigma-Aldrich) at 10 μM final concentration for 4 hours. Cells were harvested and total extracts prepared for subsequent analysis as described.

### Subcellular Localization Assay

U2OS cells were transfected either with 1,5 μg of wt ARF, or 2 μg of ARFT8A, or 1,5 μg of ARFT8D expressing plasmids. 16 hours after transfection cells were treated with DMSO or 10 μg MG132 (Sigma-Aldrich) for 5 hours.

Immunofluorescence assay was performed as described [11]. Briefly, cells were fixed with 4% paraformaldehyde (Sigma-Aldrich, Germany) for 10’ at RT. Cells were permeabilized with ice-cold 0.5% Triton X-100 for 5 min and then washed with PBS. Cells were then incubated with anti-ARF antibody (14PO2 clone, Neomarkers) for 1hr at RT, followed by incubation with a Cy3-conjugated anti-mouse antibody (ImmunoResearch Laboratory) for 1 hour at RT. DAPI (Sigma-Aldrich, Germany) staining was performed to counterstain nuclei. Coverslip were mounted with Vectashield (VectorLab) and examined under a fluorescence microscope (Nikon). For each transfection point, subcellular localizations were analysed in 100–150 cells and results plotted in graph. Histograms (% of transfected cell ± S.D.) represent the mean of at least three independent transfection experiments.

Nucleo-cytoplasmic extraction of U2OS electroporated cells was performed as previously described in Vivo et al., 2013. Equal amount of either nuclear (6 μg) or cytoplasmic extracts 30 μg) were subjected to SDS-page and Western blot as described.
